# The role of PET-CT in radiotherapy planning of solid tumours

**DOI:** 10.2478/raon-2013-0071

**Published:** 2015-03-03

**Authors:** Stasa Jelercic, Mirjana Rajer

**Affiliations:** Department of Radiotherapy, Institute of Oncology Ljubljana, Ljubljana, Slovenia

**Keywords:** positron emission therapy, radiotherapy, radiotherapy planning, tumour biology

## Abstract

**Background:**

PET-CT is becoming more and more important in various aspects of oncology. Until recently it was used mainly as part of diagnostic procedures and for evaluation of treatment results. With development of personalized radiotherapy, volumetric and radiobiological characteristics of individual tumour have become integrated in the multistep radiotherapy (RT) planning process. Standard anatomical imaging used to select and delineate RT target volumes can be enriched by the information on tumour biology gained by PET-CT. In this review we explore the current and possible future role of PET-CT in radiotherapy treatment planning. After general explanation, we assess its role in radiotherapy of those solid tumours for which PET-CT is being used most.

**Conclusions:**

In the nearby future PET-CT will be an integral part of the most radiotherapy treatment planning procedures in an every-day clinical practice. Apart from a clear role in radiation planning of lung cancer, with forthcoming clinical trials, we will get more evidence of the optimal use of PET-CT in radiotherapy planning of other solid tumours.

## Introduction

Cancer treatment has undergone major progress in the past decades. Many previously untreatable malignancies are now-days being successfully cured mostly by the combination of various treatment modalities. Radiotherapy (RT) is almost always part of them. When prescribing radical radiotherapy to the patients we need to achieve two goals; the target volume of the tissue irradiated to high-dose must encompass the entire tumour and any microscopic extensions of disease and the dose to the normal tissues should be kept as low as possible to avoid major acute and late complications. To arrive at these goals we have to combine technical improvements and clinical experiences.^1^

The most important technical improvements consist of integration of computed tomography (CT) imaging into treatment planning and introduction of computer controlled multileaf collimator system. They enable development of more efficient techniques for dose delivery, such as 3-dimensional conformal radiotherapy (3D-CRT), intensity-modulated radiotherapy (IMRT) and volumetric arc radiotherapy (VMAT).^1^,^2^ Combined with innovative in-room image-guidance systems they increase precision and accuracy of radiation delivery.^3^

For target delineation we need accurate tumour assessment. Until recently, morphologic (anatomical) imaging with CT and/or MRI scans, was the only information available in the treatment planning process.^1^,^4^,^5^ This type of imaging is unable to describe all tumour characteristics. The progress in nuclear medicine has brought an additional perspective to define the extent and characteristics of the tumour. A new concept of ‘biologic imaging’ has been coined, which provides metabolic, functional, physiological, genotyping and fenotyping information.^2^

Namely, alongside with the innovations in medical physics and nuclear medicine imaging, there has been a major leap in the understanding of tumour biology. It is now recognized that cancer is not a homogeneous ensemble of cancer cells with similar attributes, but that consists of subvolumes with very different radiobiological properties such as hypoxic areas that are known to be highly radio-resistant, regions with uncontrolled cellular proliferation.^3^

## The role of PET in tumour assessment and RT treatment planning

The integration of [18F]-fluorodeoxyglucose-positron emission tomography with computed tomography imaging (18FDG-PET-CT) has become an essential part in the evaluation of various types of malignancies.^2^,^6^ Its role has been widely accepted and confirmed in the staging process, the evaluation of response to treatment and detection of tumour recurrence.^7^–^11^ However, the role of PET-CT has been proposed and studied in some other settings, especially in the planning of radiation delivery.^5^,^7^,^12^,^13^

Positron Emission Tomography (PET) scanning has become a paradigm for molecular imaging.^6^ By administering different radiolabeled substances to the patients, we can identify biological characteristics of their tumours non-invasively.^1^ Examples include the uptake of radiolabeled misonidazole as a surrogate for some forms of hypoxia, thymidine for cell proliferation, acetate and choline for lipid metabolism, methionine for amino acid uptake and the most used and studied [F18]-luorodeoxyglucose (FDG).^14^ It is well known that many malignancies have higher metabolism and consequently uptake FDG more than surrounding normal tissues. This allows FDG-PET to image them.^1^ Today more than 95% of the molecular imaging procedures in oncology make use of FDG.^15^

In radiotherapy planning, biologic imaging is particularly useful when the patient has poorly defined target volumes, (*e.g*. brain tumour or lung cancer), or when the intent of RT is to deliver higher than standard doses of radiotherapy (called dose escalation) to the tumour in order to kill as many tumour cells as possible and damage as few as possible normal tissue cells (*e.g*. head and neck cancer, lung carcinoma, prostate carcinoma).^16^ This type of RT planning needs accurate definition of the metabolically active tumour volume and its differentiation from surrounding tissue.

In the International Atomic Energy Agency report 2006–2007 experts concluded that RT based on PET-CT can be more accurate compared to RT based on standard CT in these cases:^1^

- Imaging of lesions not apparent on CT or MRI, such as unsuspected lymph nodes or distant metastases
- Prevention of irradiation of tissues that don’t contain tumour cells, such as atelectasis in the case of lung carcinoma
- When chemotherapy is added to RT, response to it can be assessed better with PETCT, than with CT alone
- Development of “response adapted therapy” in which changes to target volumes could potentially be made during a treatment course.

Additionally, PET-CT is being studied as a replacement of conventional imaging techniques, especially in IMRT planning, which allows the delivery of non-uniform radiation intensity and non-homogeneous dose distribution inside the target volume.^17^–^19^ Imaging of biologically diverse tumour sub-volumes could potentially allow administration of different radiation doses to different tumour regions based on suspected tumour burden or radiosensitivity of the region of interest.^17^ For this focal dose escalation (inside the target) with the intent to improve the local control the terms ‘dose painting’ (2D) or ‘dose sculpting’ (3D) have been coined.^6^

In conformal 3D radiotherapy planning different volumes (anatomical units) need to be defined in order to deliver RT: gross tumour volume (GTV) includes macroscopically visible tumour and involved lymph nodes; clinical target volume (CTV) is derived from GTV by adding margins to it and includes all subclinical disease; and planning target organ (PTV) which is derived from variations in the size/position of CTV (physiological processes, tumour reduction or swelling) and the patient (weight loss, physiological processes, movements, daily set-up or technical errors).^17^ With development of biologically orientated RT, beside the search for a reliable delineation of the whole malignant lesion, the definition of biologically tumour subvolumes (BTV) is becoming another point of interest.^16^ The idea is that by selectively boosting radio-resistant areas while decreasing the doze to more susceptible zones, local tumour control rates could increase without increased side effects.^21^

When PET or PET-CT is used for RT planning, precise protocols should be followed and consistently applied.^22^ Main uncertainty in applying these protocols in every day clinical practice is tumour contouring based on PET (PET-CT). Up to now PET-based tumour contouring was mainly affected by the (investigator’s) choice of threshold. There have been some attempts to standardize lesion delineation (GTV) in FDG-PET-derived images: from arbitrary appointed threshold value as a percentage of the maximum uptake (*e.g*. 40%, 50%), threshold depending on the background signal, to defined absolute standardized uptake value (SUV) (*e.g*. 2 ± 0.4), and, the most commonly used, the visual interpretation of the PET scan.^23^–^25^ Nestle *et al*. compared different techniques of tumour contour definition by FDG-PET that lead to substantially different volumes, especially in patients with inhomogeneous tumors.^26^ Furthermore, Yu *et al*. tried to determine the cut-off value of SUV by matching the pathologic gross tumour volume from whole-mount serial section of stage I non-small cell lung cancer (NSCLC) to PET-based GTV.^27^ In this prospective study, the most appropriate threshold for tumour contour was a 3.0 absolute SUV or 31% of maximal SUV. In contrast, Devic *et al.* questioned use of various automatic delineation methods for several reasons: poor resolution of functional imaging, tumour motion, and distinct pathologic sub-types.^28^ In exchange, authors considered a possible option of biologic targeting defined by anatomical modalities (CT and MRI) and multiple biological tracers to determine tumour subvolumes with different biological characteristic and different radiation dose to obtain better tumour control.

For more accurate results, innovations in image quality and reconstruction are required in order to determine the most proficient delineation technique.

The following sections provide a review of the clinical application of PET-CT in radiation treatment planning of some common cancers.

## Lung cancer

When available PET-CT should be used to select patients with NSCLC for treatment with radical RT.^1^ Several studies have shown that inclusion of PET-CT in the staging process of locally advanced NSCLC patients alters the planned treatment in up to 30% of cases by making them ineligible for radical RT, because of distant metastasis or extensive intrathoracic disease detected by PET-CT.^29^–^31^ Gregory *et al*. reported in a prospective study that for patients with NSCLC treated with radical intent, PET-CT-based staging was significantly more powerfully correlated with overall survival than conventional imaging-based staging, across all staging and within cohorts of patients given any particular form of therapy.^30^

In comparison to conventional imaging with CT or PET alone, integrated PET-CT can distinguish malignant lesions from benign lesions with an accuracy of 82% with varying sensitivity and specificity values (from 79% to 96% and from 40% to 83%, respectively).^32^ For mediastinal node stage, the benefit of PET-CT lies especially in very high negative predictive value over 90% with the sensitivity, specificity, positive predictive value and accuracy of 73%, 80%, 78% and 87%, respectively.^33^

Evaluation of the neoadjuvant chemo-radiotherapy response before potential surgery is another possible implication of functional imaging.^34^–^36^ The survival of patients with persistent FDG uptake after radiotherapy or surgery is significantly worse than those without residual activity.^37^ A recent study published by Usmanij *et al.* revealed that the degree of early metabolic change already after the second week of chemo-radiotherapy can predict the response to treatment.^38^

Main limitation of FDG-PET as re-assessment tool is that it is not as good as in primary staging of mediastinal lymph nodes (sensitivity and specificity for detection being 63% and 85%, respectively). Currently it is not recommended as the only diagnostic tool in therapeutic decisions when restaging patients after induction therapy in stage III NSCLC.^34^

Aerts *et al*. disclosed that areas with residual FDG uptake after high dose (chemo)-radiotherapy largely overlapped with the areas of high FDG uptake locations before treatment.^39^ However, future trials should provide the basis to test if FDG uptake reflects ‘radioresistance’, by boosting high FDG uptake areas. Moreover, more specific tracer as 18F-fluoromisomidazole may be useful for dose-painting within the tumour as well.^40^

## PET-CT in the planning of lung tumour radiotherapy

Of all the common cancers, lung cancer has been most intensively studied by integrating dual imaging into the radiation treatment management of patients.^1^,^41^ There are two main reasons to use PET-CT in definitions of the volume needed to be irradiated:

- PET-CT significantly changes lymph node staging in the thorax, usually by showing more positive lymph nodes than CT
- In cases with atelectasis, PET-CT helps to define the border between tumour and atelectasis, allowing a smaller volume of lung to be treated.

**FIGURE 1.**
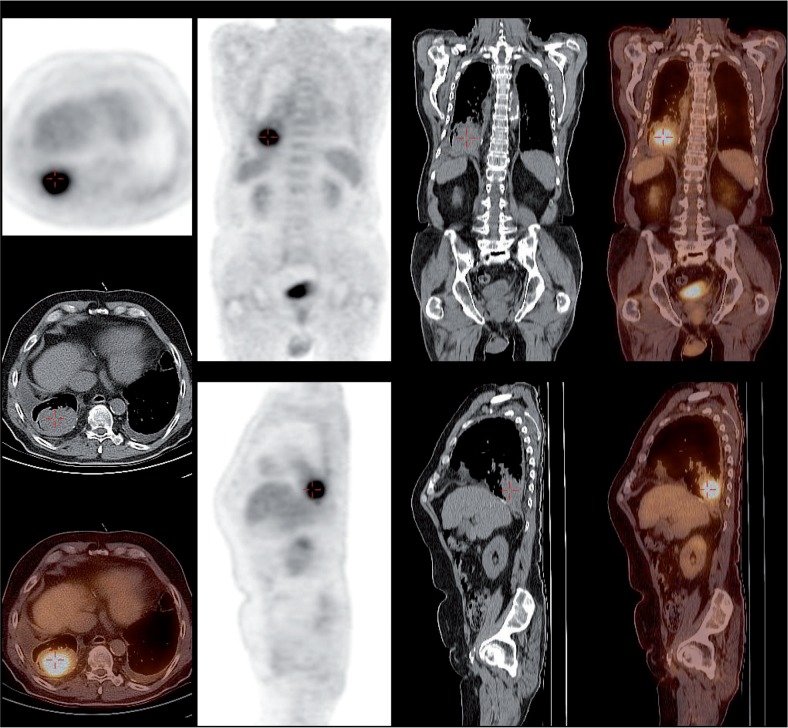
Differences in lung tumour and atelectasis seen on PET-CT.

Differences between tumour and atelectasis on PET-CT image are shown in Figure 1.

Nestle *et al.* have reviewed the results of 18 trials involving 661 patients with lung cancer that compared delineated target volumes, GTV, CTV and PTV using CT alone with the target volumes delineated using additional FDG-PET.^41^ Although the method of comparison was very different between trials, all of them came to the same conclusion that addition of FDG-PET would add essential information to CT result with significant consequences on GTV, CTV and PTV delineation. The main reasons for size modification lied in the better diagnosis of lymph node involvement and distinguishing tumour from atelectasis.^42^ Another important aspect of integrated PET-CT imaging in radiation planning was reduced interobserver delineation variability in respect to CT planning alone. The largest reduction was seen in the atelectasis region.^43^,^44^

## Head and neck cancer

Use of PET-CT can influence the treatment strategy of head and neck cancer patients in various ways. The greatest impact usually results from changes of nodal status before therapy, detection of distant metastasis and/or treatment evaluation.^4^,^45^–^47^ In patients with high risk factors for higher nodal stage or distant metastases present (poor differentiation of primary tumour, advanced T stage (T3/T4), advanced N stage (N>2), tumours arising in hypopharynx and larynx, tumours with nodes in lower neck levels (IV/IVb))^48^, the addition of pre-treatment PET imaging should be routinely used to avoid their over-treatment.^49^ Combined PET-CT showed the highest sensitivity in detecting distant metastases in comparison to only FDG-PET and CT imaging (63% *vs.* 53% and 37%).^50^

**FIGURE 2.**
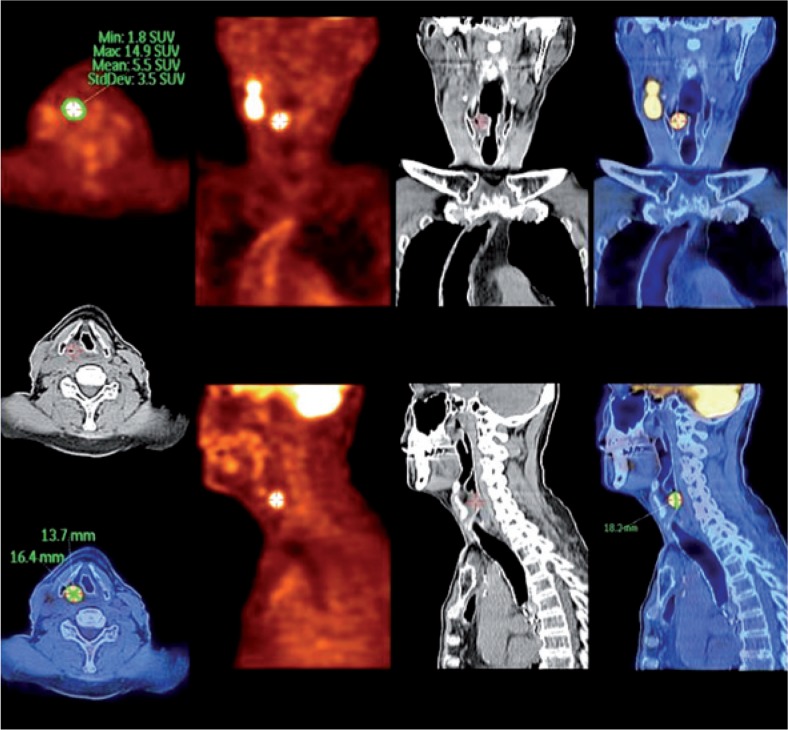
PET-CT images of 51-year old male with metastatic squamous cell carcinoma of unknown origin in cervical lymph nodes. The origin was found by PET-CT and later confirmed by a biopsy.

PET may also be of help in searching for the index tumour in patients presented with lymph node metastases of squamous cell carcinoma from unknown primary to the neck.^51^ Although detection rate of primary tumour ranges from 24%–44% as reviewed by Strojan *et al*.^52^, PET-CT should be performed when no primary lesion is suggested after thorough physical examination, indirect laryngoscopy and CT/MRI (Figure 2).^51^

The assessment of residual disease in the neck by PET-CT after chemoradiotherapy has become a standard for recognizing patients, who may avoid unnecessary neck dissection.^53^ When evaluation is performed 3 months after the end of chemoradiation, PET-CT exhibit very high negative predictive value (97–100%, as reviewed by Hamoir *et al*.)^53^, and metabolic complete response is predictive for disease- free and overall survival.^54^

## PET-CT in the planning of head and neck tumour radiotherapy

Recent studies have mainly focused on the feasibility of integrating FDG-PET with radiotherapy planning CT with the goal of enhancing tumour localization for IMRT, so the tumour coverage and normal tissue sparing can be optimized.^55^ This is an important issue when very high doses of 70 Gy or more are being administered to lesions close to radiosensitive vital structures (*e.g*. brainstem or optic chiasm).^1^ Schwartz *et al.* examined 20 head and neck cancer patients and studied the potential impact of PET-CT imaging on more tailored IMRT plans, with the exclusion of prophylactic irradiation of PET-negative regions.^55^ Their PET-CT-based IMRT planning did not suffer a geographical nodal miss when correlated with pathological examination, and dose escalation up to 75 Gy was feasible in a selected group of patients without exceeding limiting doses of critical organs.

Several published studies on head and neck cancer patients that compared RT volumes on PET images with standard CT-based planning volumes, observed significant differences in target delineation depending on the imaging technique.^1^ Daisne *et al.* compared GTVs delineated at CT, MRI and PET-CT images with resected tumour specimen.^56^ Although none of these imaging modalities was 100% accurate, the GTVs delineated at FDG-PETCT were by far the closest to the reference volume from the surgical specimens. Burri *et al.* tried to correlate the SUV to pathologic specimen size and found that fixed threshold of 40% of maximum SUV would offer the best compromise between accuracy and the risk of underestimating tumour extent.^47^

Several investigators analyzed the correlation between pre-therapeutic SUV and disease outcome and were reviewed by Inokuchi *et al*.^57^ They disclosed that not only primary tumour SUVmax, but also SUVmax of cervical lymph nodes is a prognostic factor for local control, disease-free and overall survival.^57^ Greven *et al.* reported that the changes in primary tumour SUV during and soon after completed treatment were also highly predictive of tumour recurrence compared to CT imaging alone.^58^ They concluded that adaptive therapy planning based on PET-CT may be needed in order to improve the results of the radiation therapy.

As already said, FDG uptake correlates with outcome in head and neck cancer patients and most of loco-regional recurrences occur within FDG-avid areas^59^, which would represent a reasonable target for focal dose escalation.^23^,^24^,^58^–^60^ The concept of dose painting of tumour subvolumes has been evaluated with two different methods: as dose painting by biologic image-defined contours^24^, and as dose painting by numbers, *i.e*. prescribing dose to points in the target depending of biologic signal intensity.^61^ The latter has been demonstrated to allow higher intratumour doses at similar rates of toxicity. In the study of Duprez *et al*., the median dose was escalated either to 80.9 Gy to the high-dose CTV or to 85.9 Gy to the GTV.^61^ None of the patients in the study required a treatment break and no Grade 4 acute toxicity was observed. However, which biologic characteristics of tumour and which biologic tracers are most relevant for dose painting are to be found out.

Two other promising PET tracers are 18F-fluoromisonidazole (FMISO) and 18F-fluoroazomycin arabinoside (FAZA), which provide quantitative measurements of tissue hypoxia, one of the main factors affecting treatment resistance in head and neck cancer.^62^ Rajendran *et al.* performed pretreatment FMISO-PET on 73 head and neck cancer patients and found that both the degree of hypoxia and the size of hypoxic volume measured by FMISO-PET were independent predictive factors of survival.^63^ Mortensen *et al.* found similar prognostic value of FAZA PET-CT imaging, demonstrating a significant difference in disease free survival rate in patients with non hypoxic tumours and patients with hypoxic tumours (93% and 60%, respectively, median follow up 19 months).^64^

Despite the progress in anatomical and functional imaging in defining tumour borders, meticulous, peer reviewed clinical examination cannot be replaced by any of the imaging techniques when determining mucosal extent of primary tumour.^65^ Future large clinical trials are warranted to evaluate the exact position of PET-CT in the radiotherapy treatment of head and neck cancer patients.

## Oesophageal cancer

FDG-PET-CT can provide incremental staging information compared to combined CT and endoscopic ultrasonography in up to 40% of patients and can change management in one third of patients with oesophageal carcinoma.^66^ In a systematic review by Muijs *et al.*, authors found a significant improvement of the sensitivity (93%), accuracy (92%) and negative predictive value (98%) in the assessment of locoregional lymph nodes by integrated imaging, compared to the use of CT or PET alone.^67^

There is no doubt that addition of FDG- PET in radiotherapy planning has a significant impact on target volume delineation (in 20–94% of patients), resulting in either reduction or increase in target volumes based on CT images as it is reviewed by Muijs *et al.*^67^ Trials on patients undergoing resection or endoscopic ultrasound (EUS), which is the gold standard for clinical T-staging, report that PET-based tumour length correlate well with tumour length assessed by pathologic exam or EUS. However, this does not automatically mean correct allocation of malignant tissue *in vivo*, since the FDG uptake in pathologic areas on one side can be compensated on the other side by inflammation.^67^

Some prospective studies^68^,^69^ reported about positive contribution of PET-based tumour delineation, which prevented geographic miss by identifying unsuspected malignant involvement in 30–60% of patients. However, given the poorer sensitivity of FDG-PET for primary tumour, the irradiated volume should not be reduced based on a negative FDG-PET finding in a region with suspect malignant involvement on other diagnostic investigations.^70^

Another method to utilize FDG-PET in RT treatment planning is to reduce inter-and intra-observer variability. Toya *et al*. showed significantly increased concordance rate in GTV definition (*i.e*. ratio of the intersection of the GTVs to their union) in inter- and intraobserver comparison, when incorporating FDG-PET-CT images in comparison to CT images of cervical oesophageal carcinoma.^71^ In contrast, Scheuers *et al.* found no significant effect of the additional use of FDG-PET on the interob-server variability.^69^ Several automatic (or semi-automatic) contouring methods using various thresholds have been tested, but until now have not yielded satisfactory results.^67^

Omloo *et al.* evaluated the potential prognostic value of FDG uptake in oesophageal cancer patients.^72^ Most of 31 reviewed studies showed that pretreatment FDG uptake and postneoadjuvant treatment FDG uptake, as absolute values, are predictors for survival in univariate analysis. An early decrease in FDG uptake during neoadjuvant therapy is also predictive for pathologic response and survival in most studies. However, for more reliable results, a standardized protocol for FDG–PET acquisition and reconstruction is warranted.

## Cervical cancer

T2 weighted MRI is currently the gold standard for primary tumour staging, especially in determining tumour extension in the parametria (reported accuracy of MRI is 80 to 90% compared to 60 to 69% for CT.^73^,^74^ In nodal staging PET-CT has been proven as an effective imaging technique in patients with locally advanced cervical carcinoma (*i.e.* FIGO stage is ≥IB2), with significantly better sensitivity and specificity in comparison to other morphological imaging (PET-CT sensitivity ranging from 77% to 96% for pelvic lymph nodes (PELN) and 50% to 100% for para-aortic lymph nodes (PALN), specificity from 55% to 75% for PELN and 83% to 95% for PALN, as reviewed by Magne *et al*.^75^ In spite a high negative predictive value of 92% for PALN involvement^76^, many authors still recommend a PALN dissection prior to beginning of chemoradiation, in order to avoid a possible miss of 8% of positive PALN patients.^74^

Although the presence of metastatic lymph nodes in the PELN and PALN regions does not alter the clinical stage of disease, it alters radiation treatment strategy (either by extending irradiated volume to the common iliac or para-aortic areas or by escalating irradiated dose to the involved lymph nodes).^74^ Moreover, Kidd *et al.* showed that the presence and extent (*i.e*. the most distant level of involved lymph nodes) of PET positive nodal metastases correlate well with recurrence-free and disease-free survival.^77^

Esthappan *et al*. attempted to develop a treatment plan to deliver an escalated irradiation dose to involved nodal regions without harming adjacent organ at risk.^78^ By the means of PET-CT-guided IMRT they delivered 50.4 Gy to the entire para-aortic region and 59.4 Gy to the positive para-aortic lymph nodes with acceptable irradiating dose to adjacent organs at risk. In their subsequent study, authors provided description of image acquisition, definitions of target volumes based on PET-CT and the doses prescribed to these different target volumes.^79^ Kidd *et al.* prospectively compared toxicity and clinical outcomes of cervical cancer patients treated with PET-CT-guided IMRT and conventional radiation therapy, treated to the same prescription dose. The late bowel and bladder toxicity (of Grade 3 or more) were present in only 6% in IMRT group compared to 17% in non-IMRT group. The IMRT group also showed (unexpectedly) better overall and disease specific survival and a trend towards better recurrence free survival.^80^

Several studies demonstrated that post-treatment metabolic response immediately after RT and 3 months after RT are predictive of patient outcome.^81^,^82^ Schwartz *et al.* proposed the implementation of routine post-treatment PET-CT, which could affect the approach and timing of salvage therapy of patients with cervical cancer recurrence and would also provide long-term prognostic information only 3 months after completion of therapy.^82^ Yoon *et al.* found that even earlier assessment (after receiving 54–60 Gy) of metabolic response of PELN in patients with initial PELN metastases correlates to disease free survival rate and could be a useful milestone for selecting patients who need more intense treatment.^81^

## Conclusions

Recent advances in PET-CT can broaden the radiation oncologist’s knowledge on the extent of the disease, in order to avoid missing the tumour, with consequent reduction of local control probability, and, on the other hand, avoiding the unjustified irradiation of healthy tissue. Furthermore, the correct definition of the disease stage is mandatory for selection of the most appropriate therapeutic strategy. Another important information for the radiation oncologist relates to biological characteristics of treated tumour, which could affect the response to radiotherapy and are of paramount importance for personalized and biologically oriented radiotherapy. The consensus of using PET-CT information for automated target volume delineation has not been reached yet and is awaiting further validation. PETCT can answer many questions regarding correct disease staging and its biologic characteristics, but its exact role in every-day RT treatment planning is expected to be defined in future clinical trials.
